# *catena*-Poly[[(2,2′-bipyridine-κ^2^ *N*,*N*′)cobalt(II)]-μ-oxalato-κ^4^ *O* ^1^,*O* ^2^:*O* ^1′^,*O* ^2′^]

**DOI:** 10.1107/S1600536809012690

**Published:** 2009-04-10

**Authors:** Pei-Zhou Li, Qiang Xu

**Affiliations:** a National Institute of Advanced Industrial Science and Technology (AIST), Ikeda, Osaka 563-8577, and Graduate School of Engineering, Kobe University, Nada Ku, Kobe, Hyogo 657-8501, Japan

## Abstract

In the one-dimensional title coordination polymer, [Co(C_2_O_4_)(C_10_H_8_N_2_)]_*n*_, the Co^II^ atom is coordinated in a distorted octa­hedral geometry by two N atoms from one 2,2′-bipyridine ligand and four O atoms belonging to two chelating oxalate ligands. Two neighboring Co centers are bridged by an oxalate ligand, forming a one-dimensional chain structure.

## Related literature

For general background to metal–oxalate compounds, see: Coronado *et al.* (2001 ▶); Decurtins *et al.* (1994 ▶). For related structures, see: Fun *et al.* (1999 ▶); Lin *et al.* (2006 ▶).
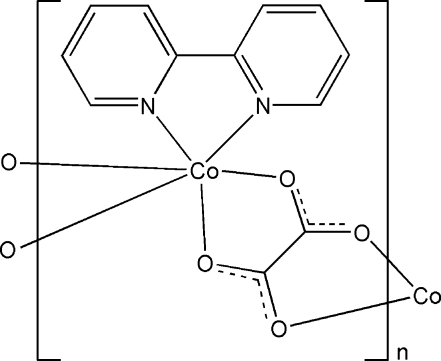


## Experimental

### 

#### Crystal data

[Co(C_2_O_4_)(C_10_H_8_N_2_)]
*M* *_r_* = 303.13
Orthorhombic, 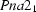

*a* = 9.2333 (18) Å
*b* = 9.2163 (18) Å
*c* = 14.101 (3) Å
*V* = 1199.9 (4) Å^3^
*Z* = 4
Mo *K*α radiation
μ = 1.44 mm^−1^
*T* = 293 K
0.16 × 0.14 × 0.08 mm

#### Data collection

Rigaku R-AXIS RAPID diffractometer
Absorption correction: multi-scan (*ABSCOR*; Higashi, 1995 ▶) *T*_min_ = 0.800, *T*_max_ = 0.892
10848 measured reflections
2692 independent reflections
1887 reflections with *I* > 2σ(*I*)
*R*_int_ = 0.064

#### Refinement

*R*[*F* ^2^ > 2σ(*F* ^2^)] = 0.052
*wR*(*F* ^2^) = 0.136
*S* = 1.09
2692 reflections
172 parameters
1 restraint
H-atom parameters constrained
Δρ_max_ = 0.38 e Å^−3^
Δρ_min_ = −0.90 e Å^−3^
Absolute structure: Flack (1983 ▶); 1283 Friedel pairs
Flack parameter: 0.03 (4)

### 

Data collection: *PROCESS-AUTO* (Rigaku, 1998 ▶); cell refinement: *PROCESS-AUTO*; data reduction: *PROCESS-AUTO*; program(s) used to solve structure: *SHELXS97* (Sheldrick, 2008 ▶); program(s) used to refine structure: *SHELXL97* (Sheldrick, 2008 ▶); molecular graphics: *PLATON* (Spek, 2009 ▶) and *ORTEP-3* (Farrugia, 1997 ▶); software used to prepare material for publication: *publCIF* (Westrip, 2009 ▶).

## Supplementary Material

Crystal structure: contains datablocks I, global. DOI: 10.1107/S1600536809012690/hy2191sup1.cif

Structure factors: contains datablocks I. DOI: 10.1107/S1600536809012690/hy2191Isup2.hkl

Additional supplementary materials:  crystallographic information; 3D view; checkCIF report

## Figures and Tables

**Table 1:** Selected bond lengths (Å)

Co1—O1	2.076 (4)
Co1—O4^i^	2.095 (4)
Co1—O2^i^	2.126 (4)
Co1—N2	2.139 (5)
Co1—O3	2.142 (4)
Co1—N1	2.146 (5)

Symmetry code: (i) 
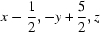
.

**Fig. 1.**
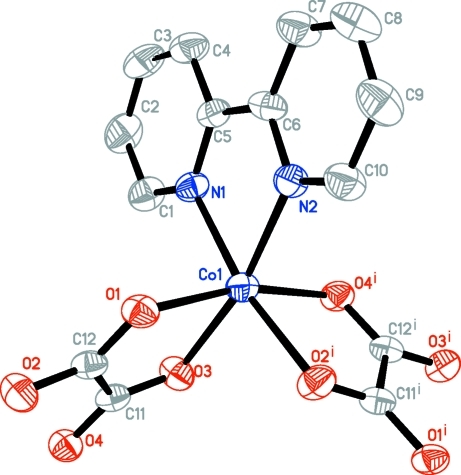
The asymmetric unit of the title compound, extended to show the Co coordination. Displacement ellipsoids are drawn at the 50% probability level. H atoms have been omitted for clarity. [Symmetry code: (i) x-1/2, -y+5/2, z.]

**Fig. 2.**
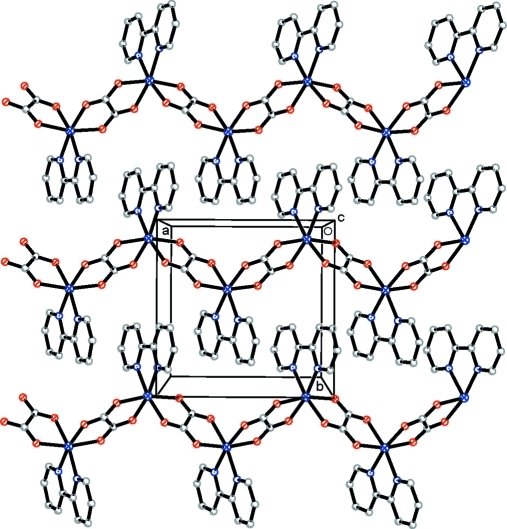
The one-dimensional chian structure of the title compound.

## supplementary crystallographic information

### Comment

The self-assembly of coordination polymers has attracted considerable attention in the past decade. This arises mainly for their various intriguing topological structures and their potential applications in material chemistry. Much research has been carried out on metal–oxalate compounds due to their interesting magnetic and optical properties (Coronado *et al.*, 2001; Decurtins *et al.*, 1994). Herein, we present the structure of the title complex, [Co(C_2_O_4_)(C_10_H_8_N_2_)]_n_, which is isomorphous to the zinc(II) complex reported by Lin *et al.* (2006) and to the iron(II) complex reported by Fun *et al.* (1999).

The title compound is an infinite one-dimensional coordination polymer. The Co^II^ atom is coordinated by two N atoms from one 2,2'-bipyridine ligand and four O atoms that belong to two oxalate dianions (Table 1) in a distorted octahedral geometry, as shown in Fig. 1. Two neighboring Co centers are bridged by an oxalate ligand, forming a one-dimensional chain structure, as shown in Fig. 2.

### Experimental

The brown plate-like single crystals of the title compound were obtained by a solvothermal reaction of cobalt chloride hexahydrate (CoCl_2_.6H_2_O, 0.5 mmol), sodium oxalate [Na_2_(C_2_O_4_), 1 mmol] and 2,2'-bipyridine (C_10_H_8_N_2_, 0.5 mmol) in a solution of CH_3_OH and H_2_O (8 ml, volume ratio, 1:1) at 423 K for 24 h.

### Refinement

H atoms were positioned geometrically and refined as riding atoms, with C—H = 0.93 Å and with *U*_iso_(H) = 1.2*U*_eq_(C).

### Crystal data

**Table:**

[Co(C_2_O_4_)(C_10_H_8_N_2_)]	*F*(000) = 612
*M**_r_* = 303.13	*D*_x_ = 1.678 Mg m^−^^3^
Orthorhombic, *P**n**a*2_1_	Mo *K*α radiation, λ = 0.71073 Å
Hall symbol: P 2c -2n	Cell parameters from 2143 reflections
*a* = 9.2333 (18) Å	θ = 3.4–26.5°
*b* = 9.2163 (18) Å	µ = 1.44 mm^−^^1^
*c* = 14.101 (3) Å	*T* = 293 K
*V* = 1199.9 (4) Å^3^	Plate, brown
*Z* = 4	0.16 × 0.14 × 0.08 mm

### Data collection

**Table:**

Rigaku R-AXIS RAPID diffractometer	2692 independent reflections
Radiation source: rotating anode	1887 reflections with *I* > 2σ(*I*)
graphite	*R*_int_ = 0.064
ω scans	θ_max_ = 27.5°, θ_min_ = 3.1°
Absorption correction: multi-scan (*ABSCOR*; Higashi, 1995)	*h* = −10→11
*T*_min_ = 0.800, *T*_max_ = 0.892	*k* = −11→11
10848 measured reflections	*l* = −18→18

### Refinement

**Table:**

Refinement on *F*^2^	Secondary atom site location: difference Fourier map
Least-squares matrix: full	Hydrogen site location: inferred from neighbouring sites
*R*[*F*^2^ > 2σ(*F*^2^)] = 0.052	H-atom parameters constrained
*wR*(*F*^2^) = 0.136	*w* = 1/[σ^2^(*F*_o_^2^) + (0.0501*P*)^2^ + 1.1682*P*] where *P* = (*F*_o_^2^ + 2*F*_c_^2^)/3
*S* = 1.09	(Δ/σ)_max_ = 0.001
2692 reflections	Δρ_max_ = 0.38 e Å^−^^3^
172 parameters	Δρ_min_ = −0.89 e Å^−^^3^
1 restraint	Absolute structure: Flack (1983); 1283 Friedel pairs
Primary atom site location: structure-invariant direct methods	Flack parameter: 0.03 (4)

### Fractional atomic coordinates and isotropic or equivalent isotropic displacement parameters (Å^2^)

**Table:**

	*x*	*y*	*z*	*U*_iso_*/*U*_eq_	
O2	0.5044 (5)	1.2574 (5)	0.3939 (3)	0.0580 (13)	
O4	0.4266 (4)	1.3874 (4)	0.2309 (3)	0.0507 (10)	
C12	0.3425 (7)	1.2932 (7)	0.2640 (4)	0.0463 (14)	
Co1	0.11792 (7)	1.08924 (7)	0.30925 (11)	0.0484 (2)	
C11	0.3876 (6)	1.2177 (7)	0.3569 (4)	0.0441 (13)	
O1	0.3038 (5)	1.1224 (4)	0.3891 (3)	0.0504 (10)	
O3	0.2237 (5)	1.2560 (5)	0.2294 (3)	0.0543 (12)	
C5	0.1633 (8)	0.7791 (7)	0.2550 (5)	0.0494 (15)	
C6	0.0832 (8)	0.7718 (7)	0.3458 (5)	0.0519 (16)	
N1	0.1983 (6)	0.9123 (6)	0.2254 (4)	0.0482 (12)	
N2	0.0502 (6)	0.9010 (5)	0.3861 (4)	0.0485 (12)	
C1	0.2749 (8)	0.9265 (8)	0.1462 (5)	0.0628 (18)	
H1	0.3012	1.0189	0.1262	0.075*	
C10	−0.0191 (8)	0.9026 (8)	0.4686 (5)	0.0630 (17)	
H10	−0.0402	0.9916	0.4964	0.076*	
C4	0.2031 (9)	0.6552 (8)	0.2047 (6)	0.066 (2)	
H4	0.1776	0.5634	0.2264	0.079*	
C9	−0.0611 (9)	0.7770 (9)	0.5146 (5)	0.074 (2)	
H9	−0.1083	0.7819	0.5728	0.088*	
C7	0.0401 (9)	0.6451 (8)	0.3887 (6)	0.069 (2)	
H7	0.0604	0.5569	0.3596	0.083*	
C2	0.3166 (9)	0.8083 (11)	0.0928 (6)	0.078 (2)	
H2	0.3686	0.8209	0.0369	0.094*	
C8	−0.0327 (10)	0.6461 (9)	0.4741 (6)	0.078 (2)	
H8	−0.0615	0.5601	0.5031	0.094*	
C3	0.2799 (10)	0.6717 (9)	0.1236 (6)	0.080 (2)	
H3	0.3077	0.5907	0.0888	0.096*	

### Atomic displacement parameters (Å^2^)

**Table:**

	*U*^11^	*U*^22^	*U*^33^	*U*^12^	*U*^13^	*U*^23^
O2	0.058 (3)	0.056 (3)	0.060 (3)	−0.010 (2)	−0.018 (3)	0.017 (2)
O4	0.057 (2)	0.046 (2)	0.049 (2)	−0.0021 (19)	−0.005 (2)	0.0123 (19)
C12	0.059 (3)	0.035 (3)	0.045 (3)	0.008 (3)	−0.003 (3)	0.002 (3)
Co1	0.0518 (4)	0.0394 (3)	0.0541 (4)	0.0005 (4)	−0.0009 (5)	−0.0032 (5)
C11	0.058 (4)	0.034 (3)	0.040 (3)	0.004 (3)	−0.007 (3)	0.001 (2)
O1	0.060 (2)	0.043 (2)	0.048 (2)	−0.0009 (19)	−0.007 (2)	0.0121 (19)
O3	0.058 (3)	0.055 (3)	0.050 (3)	−0.009 (2)	−0.014 (2)	0.009 (2)
C5	0.054 (3)	0.040 (3)	0.054 (4)	0.007 (3)	−0.004 (4)	0.000 (3)
C6	0.066 (4)	0.038 (3)	0.052 (4)	−0.007 (3)	−0.015 (4)	0.005 (3)
N1	0.057 (3)	0.044 (3)	0.044 (3)	0.006 (2)	0.005 (2)	−0.003 (2)
N2	0.053 (3)	0.046 (3)	0.046 (3)	−0.002 (2)	0.007 (3)	−0.003 (2)
C1	0.073 (4)	0.064 (4)	0.051 (4)	0.009 (4)	0.011 (4)	−0.001 (3)
C10	0.073 (4)	0.054 (4)	0.062 (4)	−0.011 (3)	0.006 (4)	−0.006 (3)
C4	0.080 (5)	0.046 (4)	0.073 (5)	0.008 (3)	−0.003 (4)	−0.013 (3)
C9	0.089 (5)	0.082 (5)	0.050 (4)	−0.023 (4)	0.013 (4)	0.004 (4)
C7	0.086 (5)	0.051 (4)	0.070 (5)	−0.013 (4)	0.006 (4)	−0.007 (4)
C2	0.083 (5)	0.090 (6)	0.062 (5)	0.015 (5)	0.022 (5)	−0.010 (4)
C8	0.102 (6)	0.062 (5)	0.071 (5)	−0.023 (4)	0.000 (5)	0.009 (4)
C3	0.094 (6)	0.063 (5)	0.082 (6)	0.027 (4)	0.006 (5)	−0.024 (4)

### Geometric parameters (Å, °)

**Table:**

O2—C11	1.252 (7)	C6—C7	1.374 (10)
O2—Co1^i^	2.126 (4)	N1—C1	1.328 (8)
O4—C12	1.254 (7)	N2—C10	1.327 (9)
O4—Co1^i^	2.095 (4)	C1—C2	1.379 (10)
C12—O3	1.248 (7)	C1—H1	0.9300
C12—C11	1.541 (6)	C10—C9	1.383 (10)
Co1—O1	2.076 (4)	C10—H10	0.9300
Co1—O4^ii^	2.095 (4)	C4—C3	1.355 (12)
Co1—O2^ii^	2.126 (4)	C4—H4	0.9300
Co1—N2	2.139 (5)	C9—C8	1.360 (11)
Co1—O3	2.142 (4)	C9—H9	0.9300
Co1—N1	2.146 (5)	C7—C8	1.379 (11)
C11—O1	1.256 (7)	C7—H7	0.9300
C5—N1	1.336 (8)	C2—C3	1.375 (12)
C5—C4	1.393 (10)	C2—H2	0.9300
C5—C6	1.480 (8)	C8—H8	0.9300
C6—N2	1.354 (8)	C3—H3	0.9300
			
C11—O2—Co1^i^	112.6 (4)	C1—N1—C5	118.7 (6)
C12—O4—Co1^i^	113.4 (4)	C1—N1—Co1	124.9 (5)
O3—C12—O4	126.0 (6)	C5—N1—Co1	116.3 (4)
O3—C12—C11	116.5 (5)	C10—N2—C6	119.1 (6)
O4—C12—C11	117.5 (5)	C10—N2—Co1	125.1 (5)
O1—Co1—O4^ii^	165.57 (14)	C6—N2—Co1	115.8 (4)
O1—Co1—O2^ii^	90.30 (17)	N1—C1—C2	122.1 (7)
O4^ii^—Co1—O2^ii^	79.14 (16)	N1—C1—H1	119.0
O1—Co1—N2	94.94 (18)	C2—C1—H1	119.0
O4^ii^—Co1—N2	95.99 (19)	N2—C10—C9	122.5 (7)
O2^ii^—Co1—N2	96.4 (2)	N2—C10—H10	118.8
O1—Co1—O3	78.61 (16)	C9—C10—H10	118.8
O4^ii^—Co1—O3	91.91 (17)	C3—C4—C5	118.4 (7)
O2^ii^—Co1—O3	92.47 (16)	C3—C4—H4	120.8
N2—Co1—O3	169.12 (18)	C5—C4—H4	120.8
O1—Co1—N1	97.18 (19)	C8—C9—C10	119.4 (8)
O4^ii^—Co1—N1	94.55 (18)	C8—C9—H9	120.3
O2^ii^—Co1—N1	169.92 (18)	C10—C9—H9	120.3
N2—Co1—N1	76.34 (17)	C6—C7—C8	121.3 (8)
O3—Co1—N1	95.6 (2)	C6—C7—H7	119.3
O2—C11—O1	125.7 (6)	C8—C7—H7	119.3
O2—C11—C12	117.1 (5)	C3—C2—C1	118.8 (8)
O1—C11—C12	117.2 (5)	C3—C2—H2	120.6
C11—O1—Co1	114.6 (4)	C1—C2—H2	120.6
C12—O3—Co1	113.1 (4)	C9—C8—C7	117.8 (7)
N1—C5—C4	122.0 (7)	C9—C8—H8	121.1
N1—C5—C6	115.6 (6)	C7—C8—H8	121.1
C4—C5—C6	122.3 (7)	C4—C3—C2	119.9 (7)
N2—C6—C7	119.8 (7)	C4—C3—H3	120.1
N2—C6—C5	115.8 (6)	C2—C3—H3	120.1
C7—C6—C5	124.3 (7)		
			
Co1^i^—O4—C12—O3	−178.1 (5)	O3—Co1—N1—C1	−5.9 (6)
Co1^i^—O4—C12—C11	3.1 (6)	O1—Co1—N1—C5	97.1 (5)
Co1^i^—O2—C11—O1	175.8 (5)	O4^ii^—Co1—N1—C5	−91.3 (5)
Co1^i^—O2—C11—C12	−4.6 (6)	O2^ii^—Co1—N1—C5	−40.5 (14)
O3—C12—C11—O2	−177.8 (7)	N2—Co1—N1—C5	3.7 (5)
O4—C12—C11—O2	1.1 (7)	O3—Co1—N1—C5	176.3 (5)
O3—C12—C11—O1	1.8 (7)	C7—C6—N2—C10	2.4 (10)
O4—C12—C11—O1	−179.2 (6)	C5—C6—N2—C10	−178.7 (5)
O2—C11—O1—Co1	178.8 (5)	C7—C6—N2—Co1	−178.0 (6)
C12—C11—O1—Co1	−0.8 (6)	C5—C6—N2—Co1	0.9 (7)
O4^ii^—Co1—O1—C11	−49.8 (10)	O1—Co1—N2—C10	81.0 (6)
O2^ii^—Co1—O1—C11	−92.5 (4)	O4^ii^—Co1—N2—C10	−89.6 (5)
N2—Co1—O1—C11	171.1 (4)	O2^ii^—Co1—N2—C10	−9.9 (6)
O3—Co1—O1—C11	0.0 (4)	O3—Co1—N2—C10	134.1 (10)
N1—Co1—O1—C11	94.3 (4)	N1—Co1—N2—C10	177.2 (5)
O4—C12—O3—Co1	179.4 (5)	O1—Co1—N2—C6	−98.6 (5)
C11—C12—O3—Co1	−1.8 (6)	O4^ii^—Co1—N2—C6	90.9 (5)
O1—Co1—O3—C12	1.1 (4)	O2^ii^—Co1—N2—C6	170.6 (5)
O4^ii^—Co1—O3—C12	170.1 (4)	O3—Co1—N2—C6	−45.5 (13)
O2^ii^—Co1—O3—C12	90.9 (4)	N1—Co1—N2—C6	−2.4 (5)
N2—Co1—O3—C12	−53.3 (13)	C5—N1—C1—C2	1.4 (11)
N1—Co1—O3—C12	−95.1 (5)	Co1—N1—C1—C2	−176.3 (6)
N1—C5—C6—N2	2.4 (7)	C6—N2—C10—C9	−1.0 (10)
C4—C5—C6—N2	−179.2 (8)	Co1—N2—C10—C9	179.5 (6)
N1—C5—C6—C7	−178.8 (8)	N1—C5—C4—C3	0.2 (11)
C4—C5—C6—C7	−0.3 (9)	C6—C5—C4—C3	−178.2 (7)
C4—C5—N1—C1	−0.9 (10)	N2—C10—C9—C8	−0.9 (12)
C6—C5—N1—C1	177.6 (6)	N2—C6—C7—C8	−2.1 (12)
C4—C5—N1—Co1	177.1 (5)	C5—C6—C7—C8	179.1 (7)
C6—C5—N1—Co1	−4.5 (7)	N1—C1—C2—C3	−1.3 (12)
O1—Co1—N1—C1	−85.1 (6)	C10—C9—C8—C7	1.2 (13)
O4^ii^—Co1—N1—C1	86.5 (6)	C6—C7—C8—C9	0.2 (13)
O2^ii^—Co1—N1—C1	137.3 (11)	C5—C4—C3—C2	0.0 (12)
N2—Co1—N1—C1	−178.4 (6)	C1—C2—C3—C4	0.5 (13)

Symmetry codes: (i) *x*+1/2, −*y*+5/2, *z*; (ii) *x*−1/2, −*y*+5/2, *z*.
